# Neurogenic bladder – concepts and treatment recommendations

**DOI:** 10.1590/S1677-5538.IBJU.2021.0098

**Published:** 2021-06-10

**Authors:** José Carlos Truzzi, Fernando Gonçalves de Almeida, Carlos Alberto Sacomani, Joceara Reis, Flávio Eduardo Trigo Rocha

**Affiliations:** 1 Universidade Federal de São Paulo - UNIFESP Departamento de Assuntos Médicos São Paulo SP Brasil Departamento de Assuntos Médicos, Universidade Federal de São Paulo - UNIFESP, São Paulo, SP, Brasil; 2 Universidade Federal de São Paulo - UNIFESP Divisão de Urologia São Paulo SP Brasil Divisão de Urologia, Universidade Federal de São Paulo - UNIFESP, São Paulo, SP, Brasil; 3 Hospital A. C. Camargo Cancer Center Divisão de Urologia São Paulo SP Brasil Divisão de Urologia, Hospital A. C. Camargo Cancer Center, São Paulo, SP, Brasil; 4 Hospital Sírio Libanês São Paulo SP Brasil Hospital Sírio Libanês, São Paulo, SP, Brasil; 5 Universidade de São Paulo - USP Departamento de Urologia São Paulo SP Brasil Departamento de Urologia, Universidade de São Paulo - USP, São Paulo, SP, Brasil

**Keywords:** Urinary Bladder, Neurogenic, Therapeutics, Urinary Sphincter, Artificial

## Abstract

**Introduction:**

Bladder and urinary sphincter malfunctioning that results from some change in the central and/or peripheral nervous system is defined as neurogenic bladder. The urinary tract symptoms that can be related to its filling, emptying, or both have a significant impact on the quality of life of individuals. The present review was based on the document prepared for the public health system in Brazil as a treatment guidelines proposal.

**Material and Methods:**

Survey questions were structured as per PICO (Population, Intervention, Control, and Outcome). Search strategies were defined and performed in the MEDLINE/Pubmed, Embase, Epistemonikos and Google Scholar databases. The selection of articles followed the evidence hierarchy concept; evidence body was identified, and the quantitative study data were extracted. The quality of evidence and grade of recommendation were qualitatively assessed according to GRADE (Grading of Recommendations, Assessment, Development and Evaluations).

**Results:**

A total of 2.707 articles were identified, with 49 of them being selected to compose the basis for this review. Neurogenic bladder treatments were classified according to their focus on filling or emptying symptoms and sub- classified in pharmacological and surgical treatments.

**Conclusion:**

Treatment guidelines are important tools for the public health system to promote the best practice when treating neurogenic bladder patients.

## INTRODUCTION

Neurogenic bladder is a term used to define bladder and urinary sphincter malfunctioning that results from some change in the central nervous system (CNS) and/or peripheral nervous system (1–3). In the pediatric population, damage often results from congenital and perinatal defects, such as cerebral palsy, spinal dysraphism, or sacral agenesis. Distinguishing between conditions producing stable damage (e.g., cerebrovascular accident, spinal cord injury, and cauda equina compression) and conditions generating progressive damage (caused by inflammatory or degenerative processes, such as dementias, Parkinson's disease, multiple sclerosis, and peripheral neuropathy) to the nervous system is also possible (2).

Patients with neurogenic lower urinary tract dysfunction have different filling, emptying, or both symptoms (2). The severity of neurogenic bladder dysfunction depends on many factors, including the neurologic lesion's location, nature, extension, and progression. Urinary tract symptoms have a significant impact on the quality of life of individuals, with urinary incontinence being the most expressive. Neurogenic urinary incontinence usually results from bladder overactivity, urethral sphincter dysfunction, or a combination of them (1).

**Figure 1:**
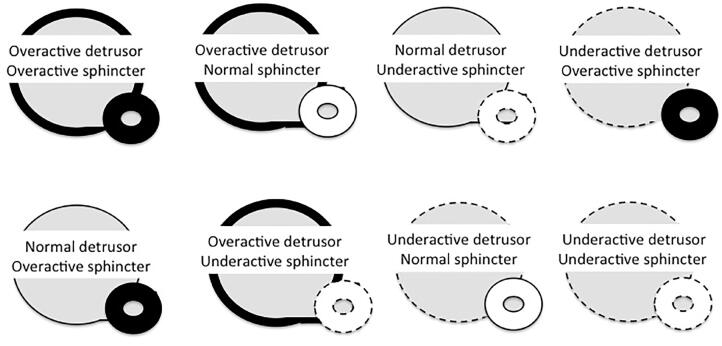
Functional classification of neurogenic voiding disorders: combination of detrusor - sphincter dysfunction secondary to damage to the innervation of the lower urinary tract that provides the basis for the current therapeutic concepts.

Figure 1 shows the Functional Classification of neurogenic voiding disorders based on detrusor functioning and urethral sphincter characteristics (4).

Medical interventions not necessarily restore the regular urinary function, but the conducts used to treat a patient's bladder dysfunction are able to improve their highly impacted quality of life. Yet, many patients will have to deal with the side effects from medications; the social and psychological consequences from the continuous use of absorbing devices; intermittent self-catheterization or permanent catheterization; urethral slings; and artificial sphincter (2).

The appropriate neurogenic bladder therapy and the successful treatment outcome are the result of an accurate diagnosis based on the patient's clinical history, physical examination, voiding diary, as well as a variety of complementary tests, including urodynamics and imaging (1).

## MATERIALS AND METHODS

The present review was based on the document prepared for the Brazilian Public Health System (SUS) as a treatment guidelines proposal.

The authors identified and defined the technologies to be considered in the recommendations document, which did not include technologies and treatments previously available at SUS. Because these previously available technologies represent the conventional clinical practice, it was decided that those treatment or follow-up recommendations would not have a defined survey question, except in cases of current uncertainties about their use, cases of disuse, or opportunities of divestment. There were no restrictions on the number of treatments to be listed.

The document exclusively covered the treatments provided to neurogenic bladder patients. Therefore, no new considerations or topics about diagnosis resources were included in the current text. The authors selected survey questions structured as per PICO (Population, Intervention, Control, and Outcome):

1. How effective and safe oxybutynin, tolterodine, solifenacin, and darifenacin are in neurogenic bladder patients?
2. How effective and safe botulinum toxin (onabotulinumtoxin A - Botox®) is in neurogenic bladder patients?
3. How effective and safe surgical treatment with a sling is in neurogenic bladder patients?
4. How effective and safe the artificial urinary sphincter is in neurogenic bladder patients?
5. Is there scientific evidence to support the use of hydrophilic catheters in adult neurogenic bladder patients?

The team of methodologists worked to design search strategies for MEDLINE/Pubmed and Embase databases. Epistemonikos and Google Scholar databases were also used to validate the findings during the search on the primary databases. The search effort was limited to articles published in English, French, and Portuguese. The terms used in the search strategy and the details are found in the Appendix.

The selection of articles followed the evidence hierarchy concept. Once the evidence body was identified, the quantitative study data was extracted. The characteristics of the selected studies were defined in order of importance for the interpretation of findings. Both study characteristics and key outcomes as defined in the survey question were extracted. The risk of bias in systematic reviews was evaluated with the use of A MeaSurement Tool to Assess systematic Reviews 2 (AMSTAR-2) in randomized clinical trials using Cochrane's bias risk tool, and in cases of observational studies, with the use of the Newcastle-Ottawa tool and the Quality Assessment of Diagnostic Accuracy Studies 2 (QUADAS-2) (5–8).

The quality of evidence and grade of recommendation were qualitatively assessed according to GRADE (Grading of Recommendations, Assessment, Development and Evaluations) criteria during the recommendation consensus meeting (9). The panel specialist's conclusions were presented in the end of the paragraph corresponding to the treatment recommendation.

## RESULTS

Two thousand seven hundred seven papers fulfilling the search criteria were identified. Incomplete texts, abstracts, and articles on repeated or duplicated topics were excluded. After a full reading, the articles to compose the database for the present review were selected according to their scientific level of evidence and relevance for the clinical practice. When two or more articles addressed the same topic, the most recent and most complete one was selected. Although many of the articles available in the literature had unarguable scientific and clinical relevance, the huge number made it impossible to include several of the publications, with 49 articles being finally selected.

Once the articles were selected, the authors reviewed them and wrote texts according to the topics for which they were designated. All summaries were presented in a joint session, and after a discussion and approval, a compilation and adjustment process led to the present text.

## DISCUSSION

### Filling dysfunction Bladder

#### Behavioral therapy

Behavioral and physical treatments may only be prescribed after the patient is evaluated by a specialist. Prescribed behavioral actions must be associated with patient, family and caregiver education about the neurogenic dysfunction. The specific physical therapy for the urinary system has restricted indication to patients with neurogenic voiding dysfunction. The Specific Physiotherapy is a restricted treatment in patients with neurogenic lower urinary tract dysfunction (NLUTD). Although the results are promising, it is important to recognize that there are no standard treatment regarding the parameters, frequency and electrodes position in different neurological disorders. Transcutaneous electrical nerve stimulation (TENS) may be effective, also demonstrate positive results by the urodynamic study findings, urinary tract symptoms and quality of life (10–13). Systematics reviews describes favorable effects of sacral and posterior tibial nerve stimulation to treat patients with NLUTD however there is a low quality of evidences. For measurable results about that treatment reliable, randomized and controlled studies are required (13). (Grade of recommendation: Weak; Quality of evidence: Low)

#### Pharmacological treatment

**Figure 2:**
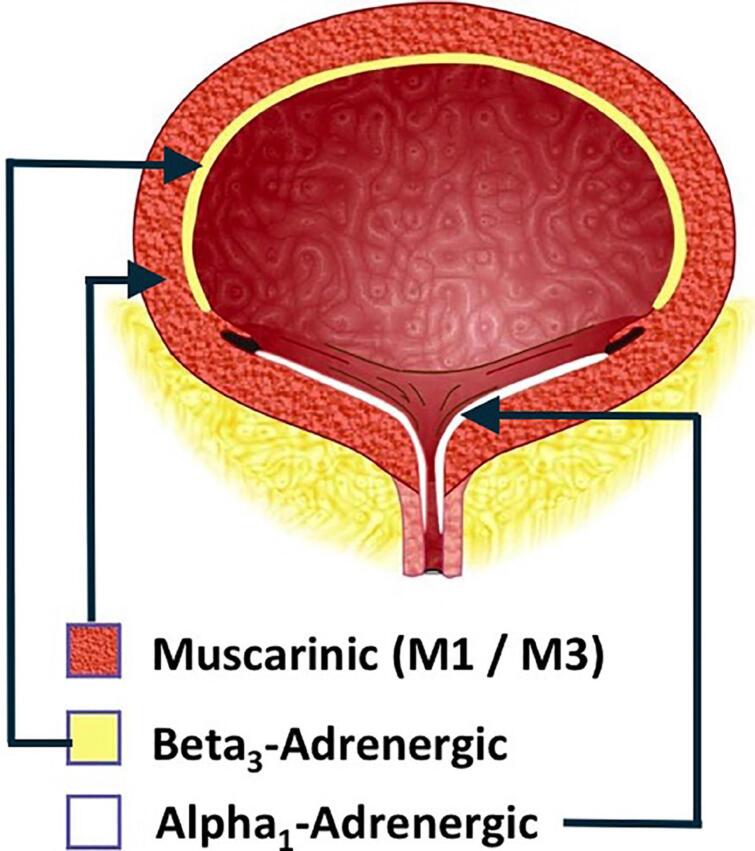
Muscarinic and adrenergic receptors of the bladder

The pharmacological treatment of the bladder factor in the neurogenic voiding dysfunction is performed in situations where detrusor overactivity is present. To date, there is not a recommendation for the use of drugs to treat neurogenic detrusor underactivity. The pharmacologic arsenal for the treatment of overactive bladder/detrusor overactivity involves antimuscarinic (or anticholinergic) drugs and beta-3 adrenergic receptor agonists (Figure-2).

#### Antimuscarinic drugs

Antimuscarinic drugs act by competitively inhibiting the muscarinic receptors (M2 and M3) in the bladder wall, which leads to detrusor muscle relaxation, intravesical pressure reduction, and as a result, increased cystometric capacity and decreased urinary incontinence secondary to detrusor overactivity (1–3, 14–16). Treatment response among patients is variable. Oftentimes, high doses or combined drugs are required to control symptoms, which lead to side effects that frequently result in treatment discontinuation (1, 17).

The main drugs currently available in Brazil are oxybutynin, tolterodine, solifenacin, and darifenacin. However, there is little scientific evidence of these drug's efficacy and safety as well as which of them would be optimal for the treatment of overactive bladder in adult neurogenic patients (1–3, 14).

There are no studies directly comparing the four antimuscarinic drugs considered here (darifenacin, oxybutynin, tolterodine, and solifenacin). Therefore, recommending a specific antimuscarinic drug is not possible. The selection must take into consideration the cost of acquisition and the safety profile for each one (1, 2, 14, 15) (Grade of recommendation: Strong; Quality of evidence: High)

#### Oxybutynin

Oxybutynin has antimuscarinic, antispasmodic, and local anesthetic effects. It has high affinity with M1 and M3 muscarinic receptors and with salivary glands, resulting in a major incidence of a dry mouth feeling with its use, which leads to a high treatment dropout rate (14–22). Oxybutynin is available as 5mg immediate-release tablets and 10mg extended-release tablets. The recommended daily dose is up to 15mg, depending on the tolerance and treatment response. Oxybutynin at ≥10mg/d doses showed a poorer adverse event profile.

#### Tolterodine

Tolterodine is a non-selective antimuscarinic drug that has higher affinity with bladder receptors than with salivary and intestinal gland receptors, and therefore, it causes a lower feeling of dry mouth and less constipation when compared with oxybutynin. The extended-release formulation shows superior efficacy with fewer adverse effects (14–16, 23, 24). Tolterodine is available as 4mg extended-release capsules. The recommended daily dose is 4mg.

#### Solifenacin

Solifenacin is an antimuscarinic drug that has higher affinity with M1 and M3 receptors and low incidence of dry mouth (14–16, 25). Solifenacin is available as 5- and 10mg extended-release tablets. The recommended daily dose is up to 10mg, depending on the tolerance and treatment response.

#### Darifenacin

Darifenacin is a more selective antimuscarinic drug for M3 receptors, showing little affinity with M1 and M2 receptors (14–16, 26). Darifenacin is available as 7.5- and 15mg extended-release tablets. The recommended daily dose is up to 15mg, depending on the tolerance and treatment response.

#### Contraindications for the use of antimuscarinic drugs

The absolute contraindications for the use of antimuscarinic drugs include urinary retention (if not included in an intermittent self-catheterization program), gastric retention, acute-angle glaucoma, and known hypersensitivity to the drug. The relative contraindications include partial obstruction of bladder emptying, renal and/or hepatic impairment, excessive use of alcohol, decreased gastrointestinal motility, constipation, and myasthenia gravis (14–16).

#### Adverse events

Randomized clinical trial systematic reviews found that oral antimuscarinics yield a significant increase of the incidence of dry mouth. Other adverse events are significantly frequent, when antimuscarinic drugs and placebo are compared - blurred vision, constipation, erythema, fatigue, profuse sweating, and urinary retention (14, 15). With regard to treatment discontinuation rate due to adverse effects, all of the evaluated antimuscarinics showed to be similar (darifenacin, solifenacin, tolterodine), except immediate-release oxybutynin that showed a higher rate of discontinuation (27, 28). Better quality-of-life results are observed in groups on extended-release than immediate-release formulations (14–16). The persistence and adherence to treatment with antimuscarinics, i.e., the time elapsed from onset to discontinuation and in which the usage dose and interval are followed as prescribed, respectively, are the lowest among the chronic-use medications for several diseases (27). Discontinuation reaches higher than 80% levels in one year (29). Cognitive changes can occur with the use of antimuscarinics. Recently, published studies have demonstrated the correlation of these drugs with cognitive disorders (30). Careful use in elderly patients and those with a history of mental illness should be considered.

#### Beta 3-adrenergic agonist

Mirabegron - the only Beta 3-adrenergic agonist representative - cannot be recommended or contraindicated in detriment to antimuscarinics as the first choice of oral drug treatment because there is no evidence supporting this recommendation yet (31–34). (Figure-2) (Grade of recommendation: Conditional; Quality of evidence: Low)

Studies evaluating mirabegron showed efficacy in controlling incontinence symptoms in patients with spinal cord injury and multiple sclerosis (31–34). However, it is worth highlighting that two of these studies were case series without a comparator group of which outcomes were only significant in relation to the baseline result. In neurogenic voiding dysfunction patients, mirabegron showed significant reductions in the number of daily micturition and reduction of incontinence episodes over 24 hours (31). It is important to stress that one of these studies only showed a significant difference in the results of micturition episodes, urgency episodes, and mean number of incontinence episodes when mirabegron was associated with desmopressin (32). No significant difference was observed in micturition results in relation to the study baseline when mirabegron was used as monotherapy. Recently, the use of mirabegron has been evaluated in patients with multiple sclerosis and spinal cord injury. There was an improvement in bladder compliance and a reduction in incontinence episodes with beta 3 adrenergic (33, 34).

The adverse events observed with the use of mirabegron are, in general, well tolerated. Arterial hypertension, tachycardia, urinary infections, dizziness, and headache are the most commonly observed adverse events following mirabegron administration (35). Patients with controlled arterial hypertension must be monitored previously to the treatment and periodically during the use of the beta 3-adrenergic medication (35, 36). The concomitant use of beta 1 and beta 2-adrenergic blockers is not contraindicated when mirabegron is administered (35, 36).

Mirabegron shows a lower discontinuation rate and a longer persistence period than antimuscarinic drugs (28). The mirabegron dose is recommended to be adjusted in patients with kidney and liver failure, and because it inhibits the enzyme CYP, caution should be taken in patients on digoxin and metoprolol (35, 36).

#### Surgical treatment Botulinum toxin

Botulinum toxin type A injection into the bladder wall is recommended in adult patients with refractory neurogenic detrusor overactivity where behavioral, physiotherapeutic, and drug therapies reveal to be ineffective or poorly tolerated (37–44) (Grade of recommendation: Strong; Quality of evidence: High).

The effects of the intravesical botulinum toxin injection have been demonstrated in both restoring detrusor stability and consequent resumption of urinary continence and protecting the upper urinary tract by avoiding the deleterious effects on kidneys caused by bladder hypertension (45–47).

#### Pre-op preparation

An urodynamic exam is indicated for evaluation of the bladder-sphincter functioning. In addition, it allows evidencing bladder compliance, urinary continence condition, and the bladder emptying phase. The upper urinary tract must be preferably evaluated using ultrasound and pertinent laboratory tests (48, 49). The general clinical evaluation and pre-op tests must be performed according to the good clinical practices. All neurogenic detrusor overactivity patients that are eligible to botulinum toxin intravesical injection, or their caregivers, must be evaluated for their manual dexterity and appropriate cognitive function and accept the possibility of intermittent self-catheterization as a bladder emptying method. One out of four patients with neurogenic detrusor overactivity will develop urinary retention and need of intermittent self-catheterization (48, 49). As many patients already use intermittent self-catheterization, this minimizes the problem of post-op urinary retention in this patient population.

#### Technique and dosage

**Figure 3:**
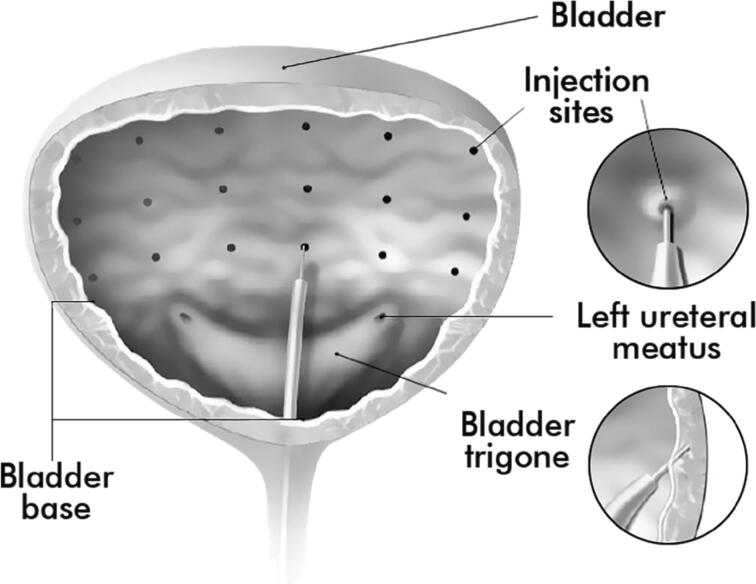
Botulinum toxin injection sites into bladder. (Image courtesy of Allergan, an AbbVie company)

Botulinum toxin is injected into the detrusor muscle through cystoscopy, and general anesthesia or sedation may be performed (50). In patients with spinal cord injury, particularly those with a lesion at the thoracic or cervical level, general anesthesia is required in order to reduce the risk of autonomic dysreflexia. For the rest of cases, it is possible to carry out the procedure under local anesthesia (50, 51). For individuals with neurogenic detrusor overactivity, the recommended dose is 200U, with the possibility of using the 300U dose at the injecting physician's discretion (52). The injection is performed into 20-30 equidistant detrusor sites (Figure-3). The trigone is usually spared due to the theoretical risk of vesicoureteral reflux, although the publications have not proven this effect yet (53). Neither have additional effects been observed when including the trigone in the injection sites (54).

#### Monitoring

The procedure is usually performed in an outpatient manner. On average, botulinum toxin effect begins two weeks after injection. Within this period, the residual volume must be monitored, either by ultrasound or bladder catheterization. In patients that do not perform catheterization, if the residual volume is higher than 150mL, establishing the intermittent self-catheterization is suggested (48, 49). There is not a fixed schedule for periodical evaluations. Botulinum toxin must be re-injected when recurrence of urinary symptoms, especially urinary incontinence is verified (on average, 9 months). The minimal interval for botulinum toxin re-injection is 12 weeks, so that anti-toxin antibody formation is not induced (48).

#### Side effects

Urinary tract infection and urinary retention are the most frequently observed adverse effects in patients with detrusor overactivity undergoing treatment with botulinum toxin (53, 55). Other reported adverse events are less frequent, and they include procedure-related pain, macroscopic hematuria and autonomic dysfunction, generalized weakness, asthenia, malaise, and flu-like symptoms (55). There is no increase of the number of adverse event cases by repeating the botulinum toxin injection (53).

#### Bladder augmentation - Enterocystoplasty

**Figure 4:**
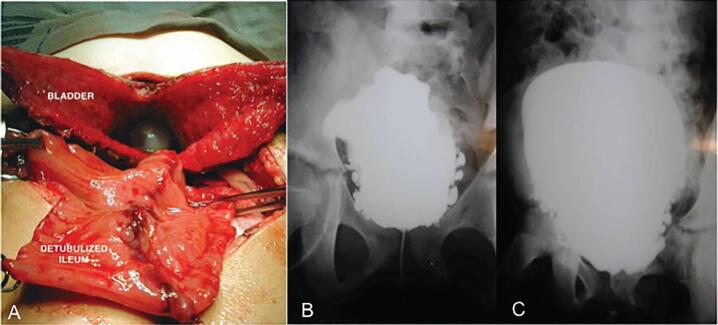
A) Enterocystoplasty procedure: open bladder (appearance of an open scallop or clam) with the Foley catheter and detubulized ileum segment being anastomosed to the bladder; B) Cystouretrography before the enterocystoplasty: trabeculated bladder with multiple diverticulae; C) Cystouretrography after the enterocystoplasty.

The surgical bladder augmentation is indicated for the treatment of neurogenic detrusor overactivity when the intravesical botulinum toxin injection fails or in cases of low bladder compliance. The objective of bladder augmentation is to increase bladder capacity and compliance, and with this, to reestablish urinary continence and protect the upper urinary tract against potential deleterious effects of high vesical pressure or chronic urinary retention (56–58). Any intestinal segment - either of the small intestine or colon - and even a portion of the stomach may be used in bladder augmentation (Figure-4). Problems related to fluid reabsorption led to the complete abandonment of the use of jejunum for this practice. Ileum is the intestinal portion of choice to surgically augment the bladder (enterocystoplasty). Cecum, colon, and sigmoid are alternatives (56). (Grade of recommendation: Strong; Quality of evidence: Moderate).

There are few studies and limited evidence on bladder auto-augmentation, and most of them are in a pediatric population. The success rate for neurogenic detrusor overactivity cases is 50%, against 92% success with enterocystoplasty (59, 60).

#### Contraindications for enterocystoplasty

The existence of intrinsic intestinal disease (e.g., Chron's disease), post-radiotherapy intestinal abnormalities, inability for or non-acceptance of intermittent self-catheterization are contraindications for enterocystoplasty. Partial kidney failure is seen as a relative contraindication, once most of neurogenic bladder-sphincter dysfunction patients maintain the renal function stable, and in some cases, they even show a reduction in the renal function decline speed following a bladder augmentation (56).

#### Pre-op preparation

An evaluation of the patient's clinical and psychiatric conditions and certification of the acceptance of intermittent self-catheterization (26–100% need intermittent self-catheterization post-operatively) are required, as well as guidance on the potential need for future interventions to achieve urinary continence (56). The upper urinary tract functioning must be verified through laboratory tests and imaging methods. Intestinal preparation must be individualized, in accordance with each patient's evacuation pattern. The current trend is not to perform an extensive intestinal preparation, except in major bowel repletion cases.

#### Post-op follow-up

There may be a demand of approximately three months to adapt to the new functional pattern. Six months following the surgery, a cystography and an urodynamic study must be performed. If there is good capacity, compliance and good bladder emptying, a urinary tract ultrasound must be performed every six months. An abdomen x-ray exam must be performed every at least two years to identify bladder stones (56). In case of ileocystoplasty, a metabolic evaluation through laboratory tests must be performed if metabolic acidosis symptoms are identified (56, 58). From the fifth year following bladder augmentation on, an annual cystoscopy helps in the early identification of malignant neoplasms (56).

#### Complications

The use of small bowel segment usually does not lead to changes in the digestive and intestinal absorptive processes. However, approximately one third of bladder-sphincter dysfunction patients undergoing enterocystoplasty progress with increased evacuation frequency, while one quarter shows post-enterocystoplasty fecal incontinence (61). The use of a more extensive intestinal segment of the ileus terminal portion may lead to a change of vitamin-B12 absorption, and as a result, anemia. Hematologic monitoring is required in the post-op follow-up (62, 63).

With regard to complications resulting from the intracavitary surgical approach, approximately 10% of patients will have intestinal obstruction due to post-ileocystoplasty adherence. Spontaneous perforation occurs in 5-10% of bladder augmentations, usually in the intestinal segment used for augmentation or in the anastomosis area with primitive bladder (60, 61, 64). The most common cause is the increase of intra-reservoir pressure secondary to drainage deficit. The diagnosis is based on clinical symptoms and imaging studies, such as ultrasound and cystography. It is worth highlighting that up to 20% of perforation cases may have a false negative result upon cystography (65). Minor leakages may be conducted conservatively with vesical drainage with a catheter. Situations with a higher urinary output to the pelvic and peritoneal cavity must be managed by surgical approach.

The formation of bladder stones is more likely to occur the longer the time elapsed since bladder augmentation. The likelihood of bladder stone formation is higher in cases in which bladder augmentation and urinary diversion are associated, such as Mitrofanoff conduit. The chances of bladder calculi recurrence within two years are 30% (65). Periodic bladder irrigation does not seem to reduce stone formation (66). When there is a urinary diversion, endoscopic handling becomes limited and the chances of residual fragments increase.

Asymptomatic bacteriuria occurs in 50-100% of patients following bladder augmentation; however, symptomatic infections are observed in 4-43% only (56, 65).

The late development of malignant neoplasms in patients with bladder augmentation due to neurogenic dysfunction is relatively rare and less frequent when compared with diversions such as ileal conduit and ureterosigmoidostomy. Periodic evaluation by cystoscopy five years from bladder augmentation and collection of oncotic cytology may be used in the diagnostic evaluation of this complication (65).

Metabolic disorders following bladder augmentation result from the absorption of substances present in the urine by the intestinal mucosa, such as water, sodium, hydrogen ion, ammonia and chloride, and the increased excretion of potassium and bicarbonate. As a result, in enterocystoplasty, there is a risk of developing hyperchloremic metabolic acidosis (67). Most patients undergoing enterocystoplasty are asymptomatic. When patients become symptomatic, oral administration of sodium bicarbonate is indicated to control the metabolic acidosis. Bone complications secondary to chronic acidosis is more frequently observed in children undergoing bladder augmentation. In adults, bone complications are less significant. Segments that are less commonly used in bladder, jejunum and colon augmentation, respectively, lead to higher water absorption and hyperchloremic metabolic alkalosis (67).

#### Sacral Neuromodulation

Sacral neuromodulation has been evaluated in patients with neurogenic bladder (68–70). Its use in this category of patients, however, is still debatable. There are case series that show some results in selected patients. However, there is a lack of randomized studies and international guidelines do not recommend its routine use. (70) (Grade of recommendation: Conditional; Quality of evidence: Low).

### Sphincter

#### Pharmacological treatment

A number of drugs, including alpha-adrenergic agonists, estrogens and tricyclic antidepressants, and duloxetine may be used to increase the resistance to urine output. However, there are no studies showing high-level evidence in neurologic patients (71). (Grade of recommendation: Weak; Quality of evidence: Low)

### Surgical treatment

#### Slings

Autologous fascial sling surgery is recommended for individuals with neurogenic stress incontinence. Sling is a treatment of choice for women with neurogenic urethral sphincter failure. Studies evaluating slings (puboprostatic, transobturator, TVT, pubovaginal) associated with bladder augmentation or associated with intradetrusor botulinum toxin injection showed favorable incontinence control results (72–79). One study comparing TVT and pubovaginal (PVS) slings showed similar failure rates in both interventions. Some quality-of-life domains were better in the PVS group than in the TVT group (79). However, due to the lack of a control group in most studies as well as the low methodological quality and small population, recommending one type of sling over the other is not possible. Thus, the aponeurotic sling is preferably recommended for women with neurogenic incontinence (77). (Grade of Recommendation: Strong [for women]; Quality of Evidence: Moderate [for women]).

A systematic review published in 2016 included 15 non-randomized studies that used male urethral slings to treat neurogenic urinary incontinence. Of the 108 men included in those studies, 26 were treated with synthetic slings. The average success rate was 58%. Complications occurred in 14% of cases, with surgical re-interventions in 7%. There was no statistically significant difference in the success rate when compared the sling technique and artificial urinary sphincter implant. However, more surgical re-interventions were necessary after implantation of artificial sphincter compared to slings (n=8 studies, mean=51±25% vs. n=14 studies, mean=7±9%; P <0.003) (80). For men with neurogenic sphincter deficiency, the use of slings seems to have modest results, and new studies with a higher level of scientific evidence to demonstrate their efficacy are needed (Grade of recommendation: Conditional [for men]; Quality of evidence: Low [for men]).

#### Artificial urinary sphincter

Artificial urinary sphincter is recommended for individuals with neurogenic urethral sphincter failure. This is the procedure of choice in males with this dysfunction. Patients using artificial sphincters - the most consistently studied is AMS 800® - usually have high rates of continence (ranging from 70-92%) post-surgery (81–96). The most frequent adverse events were infection, erosion, and re-operation (92–96). There are no studies comparing two different sphincter models nor comparing them with slings (76). Therefore, based on the current clinical practice, the use of AMS 800® sphincter is recommended in adult patients with neurogenic urethral sphincter failure (Grade of recommendation: Strong; Quality of evidence: Moderate).

It is advisable to monitor the upper urinary tract following artificial urinary sphincter surgery (e.g., performing annual ultrasound exams), as some individuals may have their bladder filling function deteriorated after treatment of neurogenic stress urinary incontinence.

### Emptying dysfunction

#### Bladder

#### Pharmacological treatment

In those patients with underactive detrusor, the use of drugs that improve detrusor contractility has already been researched. However, its use in clinical practice is controversial and there is insufficient evidence to recommend its routine use to improve bladder emptying (97). (Grade of recommendation: Weak; Quality of evidence: Low).

#### Intermittent catheterization (IC)/Intermittent self-catheterization (ISC)

Intermittent catheterization is the periodic bladder emptying method in which a catheter is introduced through the urethra or through a catheterizable conduit (such as Mitrofanoff or Monti conduits) to the bladder or urinary reservoirs. This is the procedure of choice for neurogenic lower urinary tract dysfunction with incomplete bladder emptying due to detrusor contraction deficit, or temporary or permanent urethral sphincter relaxation difficulty (98–107). In individuals with neurogenic bladder dysfunction, intermittent self-catheterization significantly reduces complications, such as urinary infection (UTI), fistulas, vesicoureteral reflux, urethral stenosis, and hydronephrosis. Furthermore, ISC leads to reduced morbidity and mortality, in addition to considerably improving the quality of life (100, 102). Hydrophilic catheters were designed to facilitate the intermittent self-catheterization technique, thereby providing patients with higher comfort and reducing the complication rates. Hydrophilic catheters, despite the cost barrier in our community, have been associated with lower rates of symptomatic urinary infection, even in the acute spinal shock phase and hematuria, when compared with PVC catheters (101, 105, 106). Systematic reviews and meta-analyses revealed favorable urinary infection and hematuria outcomes with the use of hydrophilic catheters when compared with PVC catheters (102, 106). Therefore, the use of low friction catheter is suggested in patients showing repetitive infections and with previous urethral lesions. A pharmacologic-economic study conducted in Brazil showed that the hydrophilic catheter is cost-effective for a spinal cord injury population from the perspective of the public health system (107). (Grade of recommendation: Strong; Quality of evidence: Moderate)

The frequency of the intermittent self-catheterization performance is determined by the data obtained from the voiding diary. It depends on the patient's bladder functional capacity; fluid ingestion; urodynamic parameters, such as bladder compliance; filling pressure; presence achieved in involuntary contractions; efficacy of the drugs used; presence and availability of a caregiver; etc. It is important that the drained volume is not superior to 400mL and must be regularly verified (108, 109). Some factors may limit the performance of intermittent self-catheterization, such as obesity (mainly among women), urethral lesions (diverticula and stenosis), motor sequels, tremor or manual difficulty, cognitive impairment, lower limb hypertonia, hip prosthesis, neuropathic pain, etc.

Despite the controversies, the use of prophylactic antibiotics is not recommended, once there is no evidence that it may reduce the incidence of symptomatic urinary infection episodes, although it reduces the incidence of asymptomatic bacteriuria. However, asymptomatic bacteriuria should not be treated, except when the patient undergoes surgical or endoscopic handling (102, 105).

### Intermittent self-catheterization complications

Intermittent self-catheterization is not free of risks and complications. The most commonly seen complications include urinary infections, bleeding upon handling, and urethral lesions (108).

The most frequent complication from intermittent self-catheterization is urinary tract infection (UTI). The prevalence of UTI associated with intermittent self-catheterization is highly variable in the Urology literature. This is due to the different criteria used (109–111). Case series with long-term follow-ups show that 42% of patients will have recurrent or persistent UTI (112). It should be emphasized that the treatment of UTI should only be carried out when symptoms are present (113). Intravesical instillation of antibiotics after catheterization and the use of prophylactic low-dose oral antibiotic have been the subject of some studies; however, the results are conflicting (114–116). The use of oral ascorbic acid only seems to be helpful in association with antimicrobial agents (117).

Urethral trauma with the presence of bleeding is often observed on the onset of intermittent self-catheterization, but it may persist in up to 30-60% of patients in late phases (118–120). Mucosa injury with false passage is also a frequent complication, which may occur due to the presence of urethral stenosis, detrusor-sphincter dyssynergia and increased prostatic volume. Urethral stenosis is a late complication - on average, five years after the onset of intermittent self-catheterization (119). The low resistance caused by the catheter surface with hydrophilic coating has been related to the prevention of urethral complications with significant reduction of hematuria episodes (118).

### Sphincter

#### Pharmacological treatment

Alpha-blockers may be initially used to reduce sphincter resistance and avoid autonomic dysreflexia, although their use is controversial, and their results are limited (121). (Grade of recommendation: Weak; Quality of evidence: Low)

#### Surgical treatment - Sphincterotomy

Sphincterotomy is one of the options to treat the incomplete bladder emptying in individuals with neurogenic bladder and should be considered when intermittent self-catheterization is not an option, particularly when there is a risk of upper urinary tract injury (122–126). (Grade of recommendation: Strong, Quality of evidence: Moderate)

A randomized study and a prospective cohort compared sphincterotomy with urethral stent and urethral balloon. Sphincterotomy showed significant results in reducing the voiding pressure and residual post-voiding volume three, six, and 12 months from the procedure in relation to the pre-procedure period. There was no significant difference for micturition outcomes between the groups (122, 123). However, the hospitalization time, surgery duration, and post-procedure bleeding were significantly higher in the sphincterotomy group (122).

This technique is contraindicated for both women and men with bladder acontratility or unable to adapt to a urine collecting system and it has the potential to limit human reproduction (male factor). Studies evaluating the urethral sphincterotomy showed high success rates in reducing hydronephrosis or bilateral reflux; urinary infection; autonomic dysreflexia; increased bladder emptying; reduction of lost detrusor pressure; and reduction of voiding pressure (124–126). However, this procedure leads to some complications such as hematuria, bacteremia, recurrent urinary infection, high residual volume, and autonomic dysreflexia, in addition to failures such as incomplete sphincterotomy, perineal spasticity, colon sclerosis; re-operation; urethral stenosis, and smooth sphincter dyssynergia (124–126).

#### Monitoring Neuro-Urological dysfunctions

Neuro-urological disorders are often unstable, and their symptoms may vary considerably, even within a relatively short period. For this reason, a regular follow-up is required. The main problems relative to neurogenic lower urinary tract dysfunctions are kidney failure, ureterohydronephrosis, recurrent urinary infection, and impaired quality of life due to incontinence and bladder emptying difficulty. Patient's monitoring has the purpose of avoiding the frequent infections, impeding renal lesion, and improving the patient's quality of life. Depending on the type of underlying neurological pathology and the current symptom stability, the interval between initial and control investigations may vary; in many cases, it should not exceed one to two years. In high-risk patients (ex: high intravesical pressure), this interval should be shorter (1–3).

Measuring blood creatinine and calculating the glomerular filtration (GF) rate yields a reasonable estimate of the renal function, with low cost. Creatinine clearance provides a more accurate evaluation, but it involves a 24-hour urine collection to estimate creatinine excretion. Special care should be taken in incomplete collection cases, as it may result in underestimation of the renal function. GF rate is more accurately obtained with renal scintigraphy, which is especially recommended when a low renal function is found and in high-risk patients (3). Urine test does not need to be a routine examination; it should be especially guided by the patient's symptoms (1).

The upper urinary tract must be monitored by ultrasound at regular intervals - every six months in high-risk patients (1).

Urodynamic exam must be performed in patients with lower urinary tract symptoms, particularly in cases where there is a risk of renal complications. The exam may be repeated depending on risk factors that might reflect on the upper urinary tract functioning (1, 2, 127, 128). It is a fact that the indication for a urodynamic evaluation must be made according to the good medical practice criteria when the symptoms do not allow a clear diagnosis or when the empiric treatment fails, as well as in cases where more invasive treatments are needed (3, 129, 130).

It is reasonable that any clinical changes or changes in the control tests require investigation and specialized, targeted treatment. However, we lack studies with high level of evidence on this topic, and each recommendation must be seen on an individual basis (1–3, 131). (Grade of recommendation: Strong, Quality of evidence: Low).

## APPENDIX 1 SEARCH STRATEGY

### Question 1: How effective and safe oxybutynin, tolterodine, solifenacin, and darifenacin are in neurogenic bladder patients?

#### 1) Search strategy

MEDLINE / Pubmed:

((“Urinary Bladder, Neurogenic”[Mesh] OR Neurogenic Urinary Bladder OR Neurogenic Bladder)) AND (((((“oxybutynin” [Supplementary Concept] OR oxybutynin)) OR (“Tolterodine Tartrate”[Mesh] OR Tolterodine)) OR (“Solifenacin Succinate”[Mesh] OR Solifenacin)) OR (“darifenacin”[Supplementary Concept] OR darifenacin))

Access date: 27/Oct/2017

**Total: 254 references**

EMBASE:

(‘neurogenic bladder’/exp OR ‘neurogenic bladder’ OR ‘neurogenic urinary bladder’) AND ((‘oxybutynin’/exp OR ‘oxybutynin’ OR ‘tolterodine’/exp OR ‘tolterodine’ OR ‘solifenacin’/exp OR ‘solifenacin’ OR ‘darifenacin’/exp OR ‘darifenacin’) AND [embase]/lim)

Access date: 27/Oct/2017

### Total: 574 references

#### 2) Selection of evidence

Only randomized clinical trials and systematic reviews of randomized controlled trials, which compared the antimuscarinics tolterodine, solifenacin, oxybutynin and darifenacin with placebo, with each other or with other antimuscarinics, were considered eligible.

828 references were retrieved through the search strategies (254 Medline and 574 Embase). 133 duplicates were removed. After removing duplicates, 625 references were evaluated by reading titles and abstracts. 590 references were excluded, leaving 35 references for the complete reading. After full reading 28 studies were excluded: two systematic reviews without meta-analysis; a systematic review with meta-analysis, but with incomplete quantitative analysis and most meta-analyzes with only one study; two clinical studies conducted in patients with non-neurogenic overactive bladder and 23 observational studies. Thus, seven randomized clinicians (23, 25, 17–19, 21, 22), retrieved by the search above, and two more randomized clinical studies retrieved by manual search (20, 24), totaling 9 references, were included.

### Question 2: How effective and safe botulinum toxin (onabotulinumtoxin A - Botox®) is in neurogenic bladder patients?

#### 1) Search strategy

MEDLINE / Pubmed:

(((((“Urinary Bladder, Neurogenic”[Mesh] OR “Neurogenic Urinary Bladder” OR “Bladder, Neurogenic” OR “Neurogenic Bladder” OR “Urinary Bladder Neurogenic Dysfunction” OR “Neurogenic Dysfunction of the Urinary Bladder” OR “Neurogenic Urinary Bladder Disorder” OR “Neuropathic Bladder” OR “Urinary Bladder Disorder, Neurogenic” OR “Bladder Disorder Neurogenic” OR “Neuaogenic Bladder Disorders” OR “Neurogenic Bladder Disorder” OR “Urinary Bladder Neurogenesis” OR “Neurogenesis, Urinary Bladder” OR “Bladder Neurogenesis” OR “Neurogenesis, Bladder” OR “Neurogenic Urinary Bladder, Atonic” OR “Neurogenic Bladder, Atonic” OR “Atonic Neurogenic Bladder” OR “Neurogenic; Urinary Bladder, Spastic” OR “Neurogenic Bladder, Spastic” OR “Spastic Neurogenic Bladder” OR “Neuro genic Urinary Bladder, Uninhibited” OR “Neurogenic Bladder, Uninhibited” OR “Uninhibited Neurogenic Bladder”)) AND (“Botulinum Toxin Type A”[Mesh] OR “Botulinum Toxins”[Mesh] OR”Clostridium Botulinum Toxin Type A” OR “Clostridium botulinum A Toxin” OR “Botulinum A Toxin” OR “Toxin, Botulinum A” OR “Botulinum Neurotoxin A” OR “Neurotoxin A, Botulinum” OR “Dysport “ OR “Lasa Brand of Botulinum A Toxin” OR “Speywood Brand of Botulinum A Toxin” OR “Ispen Brand of Botulinum A Toxin” OR “Oculinum” OR “Botox” OR “Merz Brand of Botulinum A Toxin” OR “Allergan Brand of Botulinum A Toxin” OR “Toxins, Botulinum “ OR”Botulinum Toxin” OR “Toxin, Botulinum” OR “Clostridium botulinum Toxins” OR “Toxins, Clostridium botulinum” OR “botulinum Toxins, Clostridium” OR “ Botulin”)) AND (((randomized controlled trial[pt] OR control led clinical trial[pt] OR randomized[tiab] OR placebo[tiab] OR (drug therapy[sh] OR randomly[tiab] OR trial[tiab] OR groups[tiab] NOT (animals [mh] NOT humans [mh])))

### Total: 423 references

Access date: 23/11Nov/2017

EMBASE:

(‘botulinum toxin type a’/exp OR ‘botulinum toxin type a’ OR ‘botulinum toxins’/exp OR ‘botulinum toxins’ OR ‘clostridium botulinum toxin type a’/exp OR ‘clostridium botulinum toxin type a’ OR ‘clostridium botulinum a toxin’/exp OR ‘clostridium botulinum a toxin’ OR ‘botulinum a toxin’/exp OR ‘botulinum a toxin’ OR ‘toxin, botulinum a’ OR ‘botulinum neurotoxin a’/exp OR ‘botulinum neurotoxin a’ OR ‘neurotoxin a, botulinum’ OR ‘dysport’/exp OR ‘dysport’ OR ‘lasa brand of botulinum a toxin’ OR ‘speywood brand of botulinum a toxin’ OR ‘ispen brand of botulinum a toxin’ OR ‘oculinum’/exp OR ‘oculinum’ OR ‘botox’/exp OR ‘botox’ OR ‘merz brand of botulinum a toxin’ OR ‘allergan brand of botulinum a toxin’ OR ‘toxins, botulinum’ OR ‘botulinum toxin’/exp OR ‘botulinum toxin’ OR ‘toxin, botulinum’ OR ‘clostridium botulinum toxins’ OR ‘toxins, clostridium botulinum’ OR ‘botulinum toxins, clostridium’ OR ‘botulin’/exp OR ‘botulin’) AND (‘urinary bladder, neurogenic’/exp OR ‘urinary bladder, neurogenic’ OR ‘neurogenic urinary bladder’ OR ‘bladder, neurogenic’/exp OR ‘bladder, neurogenic’ OR ‘neurogenic bladder’/exp OR ‘neurogenic bladder’ OR ‘urinary bladder neurogenic dysfunction’ OR ‘neurogenic dysfunction of the urinary bladder’ OR ‘neurogenic urinary bladder disorder’ OR ‘neuropathic bladder’/exp OR ‘neuropathic bladder’ OR ‘urinary bladder disorder, neurogenic’ OR ‘bladder disorder, neurogenic’ OR ‘neurogenic bladder disorders’ OR ‘neurogenic bladder disorder’ OR ‘urinary bladder neurogenesis’ OR ‘neurogenesis, urinary bladder’ OR ‘bladder neurogenesis’ OR ‘neurogenesis, bladder’ OR ‘neurogenic urinary bladder, atonic’ OR ‘neurogenic bladder, atonic’ OR ‘atonic neurogenic bladder’ OR ‘neurogenic urinary bladder, spastic’ OR ‘neurogenic bladder, spastic’ OR ‘spastic neurogenic bladder’ OR ‘neurogenic urinary bladder, uninhibited’ OR ‘neurogenic bladder, uninhibited’ OR ‘uninhibited neurogenic bladder’)

### Total: 762 references

Access date: 23/Nov/2017

#### 2) Selection of evidence

The search for evidence resulted in 1185 references (423 on MEDLINE and 762 on EMBASE). Of these, 122 were excluded because they were duplicated. One thousand and sixty-three references were screened by reading the title and abstracts, of which eighty-two references had their full texts evaluated for confirmation of eligibility. As an inclusion criterion, priority was given to systematic reviews with meta-analysis of randomized controlled trials and primary studies such as comparative randomized controlled trials. Comparative randomized controlled trials not included in systematic reviews were included.

73 studies were excluded because they were narrative reviews, letters to the editor, old systematic reviews for which there are already updates and clinical trials contained in included systematic reviews or because they used a type of botulinum toxin other than onabotulinumtoxinA.

Nine studies were included: three systematic reviews with meta-analysis (39, 40, 44) and six randomized clinical trials (37, 38, 41, 42, 43, 45).

### Question 3: How effective and safe surgical treatment with a sling is in neurogenic bladder patients?

#### 1) Search strategy

MEDLINE / Pubmed:

((“Suburethral Slings”[Mesh] OR sling surgery OR sling OR Suburethral Slings OR Midurethral Sling)) AND (“Urinary Bladder, Neurogenic”[Mesh] OR Neurogenic Urinary Bladder OR Neurogenic Bladder)

Total: 117 references

Access date: 27/Oct/2017

EMBASE:

((“Suburethral Slings”[Mesh] OR sling surgery OR sling OR Suburethral Slings OR Midurethral Sling)) AND (“Urinary Bladder, Neurogenic”[Mesh] OR Neurogenic Urinary Bladder OR Neurogenic Bladder)

### Total: 175 references

Access date: 27/Oct/2017

#### 2) Selection of evidence

The search for evidence in the MEDLINE (via PubMed) and Embase databases resulted in 292 references (117 in MEDLINE and 175 in Embase). Of these, 57 were excluded because they were duplicated. Two hundred and thirty-five references were screened by reading the title and abstracts, of which 46 had their full texts evaluated for confirmation of eligibility. Among the excluded studies, 1 was a non-systematic review, 1 was an editorial, 8 case series in pediatric patients, 4 were not treating sling, 4 clinical trials that performed sling in less than 5 patients, 2 clinical trials that included patients with post-incontinence prostatectomy, 1 study that excluded patients with neurogenic bladder, 2 studies that did not clarify the cause of incontinence and 15 non-comparative retrospectives. The inclusion criteria of the studies consisted of evaluating prospective studies that addressed the use of the technique of interest in patients with neurogenic bladder. Eight studies (8 publications) (72–76, 78–80) were included.

### Question 4: How effective and safe the artificial urinary sphincter is in neurogenic bladder patients?

#### 1) Search strategy

Medline / PubMed:

(((“Urinary Sphincter, Artificial”[Mesh] OR Artificial Urinary Sphincter OR artificial sphincter))) AND (“Urinary Bladder, Neurogenic”[Mesh] OR Neurogenic Urinary Bladder OR Neurogenic Bladder)

Access date: 27/Oct/2017

### Total: 190 references

EMBASE:

(‘neurogenic bladder’/exp OR ‘neurogenic bladder’ OR ‘neurogenic urinary bladder’) AND ((‘bladder sphincter prosthesis’/exp OR ‘bladder sphincter prosthesis’ OR ‘artificial urinary sphincter’/exp OR ‘artificial urinary sphincter’ OR ‘artificial sphincter’/exp OR ‘artificial sphincter’) AND [embase]/lim)

Access date: 27/Oct/2017

### Total: 173 references

#### 2) Selection of evidence

The search in the databases resulted in 363 references (190 on MEDLINE and 173 on EMBASE). After removing the duplicates, 280 references were screened by reading the title and abstract. Studies in the pediatric population, studies in the population with non–neurogenic urinary incontinence, case series with less than 10 patients, and case reports were excluded. Twenty-nine publications had their texts evaluated in full, to confirm eligibility, with 28 non-comparative studies and a systematic review. Of these, twelve were excluded for the following reasons: they evaluated the Adjustable Compressive Therapy device (n = 2); only a summary was available, without enough information to answer the research question (n = 3); included pediatric population (n = 2); evaluated intraurethral prosthesis (n = 1); included mixed population (urinary incontinence of different etiologies), with no information on efficacy and safety for the population of interest (n = 4).

The systematic review, without meta-analysis, published in 2014, recovered only 54 in its search (not comprehensive) on various surgical treatments in patients with neurogenic bladder and included only eight studies evaluating artificial sphincter, with a poor report on the studies. Three of these studies evaluated the pediatric population and one included a case series with less than 10 patients. So, it was decided to exclude the review and include the primary studies eligible for this research question. In the end, 13 studies (16 publications) were considered eligible to answer the research question (81–96).

### Question 5: Is there scientific evidence to support the use of hydrophilic catheters in adult neurogenic bladder patients?

#### 1) Search strategy

MEDLINE / Pubmed:

(((hydrophilic catheter* OR hydrophilic-coated catheter* OR hydrophilic coated catheter* OR hydrophilic catheterization))) AND (“Urinary Bladder, Neurogenic”[Mesh] OR Neurogenic Urinary Bladder OR Neurogenic Bladder)

Access date: 27/Oct/2017

### Total: 23 references

EMBASE:

(‘neurogenic bladder’/exp OR ‘neurogenic bladder’ OR ‘neurogenic urinary bladder’) AND ((‘hydrophilic catheter’ OR ‘hydrophilic coated catheter’ OR ‘hydrophilic-coated catheter’ OR ‘hydrophilic catheterization’) AND [embase]/lim)

Access date: 27/Oct/2017

### Total: 16 references

#### 2) Selection of evidence

The search for evidence resulted in 39 references (23 on MEDLINE and 19 on EMBASE). Of these, 11 were excluded because they were duplicated. Twenty-eight references were screened by reading the title and abstracts, of which fifteen references had their full texts evaluated for confirmation of eligibility.

As an inclusion criterion, systematic reviews with meta-analysis of randomized clinical trials and primary studies of the type randomized clinical trials were prioritized. As heterogeneity was observed between the studies and the small number of studies included in the meta-analyzes of the relevant outcomes for the research question, we chose to include the randomized clinical trials present in the systematic reviews that specifically addressed the study population.

In total, 11 studies were excluded: 1) one study did not provide sufficient or incomplete information; 2) four studies included interventions of no interest to study; 3) two studies included a pediatric population; 4) a congress summary reference had made the full study available; 5) three studies were observational; 6) two reviews were narrative. The manual search found three systematic reviews with meta-analyzes and two randomized clinical trials included in the reviews. Two randomized clinical trials were included in the search. Thus, four randomized clinical trials present in both the performed and manual searches, as well as in systematic reviews, specifically addressed the study population and were included. In total, seven studies were considered eligible (99, 101–106).
